# Supplementary material for: Acceptability and feasibility of digital adherence technologies for drug-susceptible tuberculosis treatment supervision: A meta-analysis of implementation feedback
Source: PLOS Digit Health. 2023 Aug 15;2(8):e0000322. doi: 10.1371/journal.pdig.0000322 (PMC10426983; doi:10.1371/journal.pdig.0000322)
Supplement: S1 Table — (DOCX) [file pdig.0000322.s001.docx]

**S1 Table: Constructs assessed in survey administered to people with TB by COM-B category**

| **TDF construct** | **Statement** | **Mean score (95% CI)** | |
| --- | --- | --- | --- |
|  |  | **99DOTS (N=1017)** | **evriMED (N=273)** |
| **Capability** | |  |  |
| Knowledge | I know how to use DAT | 4.59 (4.54, 4.64) | 4.90 (4.85, 4.95) |
| Memory, attention, and decision processes | The reminders I receive on my phone help me to remember to take my TB medicines | 4.40 (4.34, 4.46) | 4.25 (4.09, 4.42) |
| **Opportunity** | |  |  |
| Social influences | I am worried that using DAT makes it more likely others will find out I have TB (reversed)  I am comfortable using DAT in front of other people  I am comfortable using DAT outside of my home  I will recommend using DAT to my family or friends if they have TB  DAT helps me feel more connected to my health workers | 4.05 (3.97, 4.14)  3.75 (3.66, 3.84)  4.24 (4.18, 4.31)  4.63 (4.59, 4.68)  4.03 (3.94, 4.12) | 3.43 (3.24, 3.62)  3.56 (3.38, 3.74)  3.45 (3.26, 3.65)  4.75 (4.67, 4.84)  4.55 (4.44, 4.66) |
| Environmental context and resources | It takes me too much time to take my TB medicines and call DAT every day (reversed)  I make fewer trips to the health clinic for my TB treatment because I am using DAT | 4.30 (4.21, 4.38)  4.48 (4.43, 4.54) | 4.67 (4.56, 4.77)  3.89 (3.72, 4.06) |
| **Motivation** | |  |  |
| Optimism | Using DAT will help me to complete my TB treatment  Using DAT while taking TB medicines will help me get healthy | 4.65 (4.61, 4.70)  4.62 (4.58, 4.66) | 4.41 (4.29, 4.53)  4.31 (4.18, 4.44) |
| Reinforcement | The DAT packaging makes it easy for me to remember what to do | 4.61 (4.57, 4.65) | 4.45 (4.32, 4.58) |
| Emotion | I am concerned about the privacy of my health information collected by the DAT system | 4.18 (4.10, 4.27) | 3.36 (3.16, 3.55) |

CI: confidence interval, COM-B: Capability Opportunity Motivation Behavior; TDF: Theoretical Domains Framework; DAT: digital adherence technology; TB: tuberculosis
