# Supplementary material for: Acceptability and feasibility of digital adherence technologies for drug-susceptible tuberculosis treatment supervision: A meta-analysis of implementation feedback
Source: PLOS Digit Health. 2023 Aug 15;2(8):e0000322. doi: 10.1371/journal.pdig.0000322 (PMC10426983; doi:10.1371/journal.pdig.0000322)
Supplement: S2 Table — (DOCX) [file pdig.0000322.s002.docx]

**S2 Table: Constructs assessed in survey administered to health care workers, by COM-B category**

| **TDF construct** | **Statement** | **Mean score (95% CI)** | |
| --- | --- | --- | --- |
|  |  | **99DOTS (N=70)** | **evriMED (N=20)** |
| **Capability** | |  |  |
| Knowledge | I understand the DAT system and how it works  I received adequate training to use DAT | 4.89 (4.76, 5.00)  4.84 (4.73, 4.96) | 4.85 (4.67, 5.00)  5.00 (5.00, 5.00) |
| Memory, attention and decision processes | I remember to check the adherence data available in DAT when patients come in for refill visits  The reminders I receive from DAT help me to remember to check on patients who are not taking their medicines | 4.87 (4.79, 4.95)  4.91 (4.84, 4.99) | 4.85 (4.67, 5.00)  4.65 (4.30, 5.00) |
| Effort Expectancy* | It is easy for me to explain how to use DAT to my patients  It is easy for me to identify which patients are not taking their TB medicine using DAT | 4.97 (4.93, 5.00)  4.84 (4.76, 4.93) | 4.90 (4.76, 5.04)  4.40 (3.96, 4.84) |
| **Opportunity** | |  |  |
| Social influence^ | My patients like using DAT | 4.77 (4.66, 4.88) | 4.65 (4.42, 4.88) |
| Effort Expectancy* | It is easy for me or my co-workers to contact patients who have not taken their TB medicines  Using DAT helps to reduce my workload | 4.84 (4.76, 4.93)  4.73 (4.58, 4.87) | 4.25 (3.82, 4.68)  3.75 (3.23, 4.27) |
| **Motivation** | |  |  |
| Belief about consequences | Using DAT improves the care I provide to my patients  DAT adherence data helps me provide better support and counseling to my patients | 4.91 (4.85, 4.98)  4.96 (4.91, 5.00) | 4.80 (4.61, 4.99)  4.70 (4.48, 4.92) |
| Trust factor* | I believe that DAT data accurately reflects if my patients took their TB medicines or not | 4.70 (4.48, 4.92) | 3.90 (3.33, 4.47) |
| Purchase intention* | I would recommend using DAT to my patients | 5.00 (5.00, 5.00) | 4.75 (4.54, 4.96) |

*Key construct of UTAUT

^Both a key construct of UTAUT and a TDF domain

CI: confidence interval, COM-B: Capability Opportunity Motivation Behavior; TDF: Theoretical Domains Framework; UTAUT: Unified Theory of Acceptance and Use of Technology; DAT: digital adherence technology; TB: tuberculosis
