# Supplementary material for: Acceptability and feasibility of digital adherence technologies for drug-susceptible tuberculosis treatment supervision: A meta-analysis of implementation feedback
Source: PLOS Digit Health. 2023 Aug 15;2(8):e0000322. doi: 10.1371/journal.pdig.0000322 (PMC10426983; doi:10.1371/journal.pdig.0000322)
Supplement: S3 Table — (DOCX) [file pdig.0000322.s003.docx]

**S3 Table: Characteristics of studies included in the meta-analysis**

| **Country** | **DAT** | **TB** | **Study Period** | **Data Collection** | **Population** | **Project Summary** |
| --- | --- | --- | --- | --- | --- | --- |
| Bangladesh | 99DOTS | DS-TB | 2019-2020 | 09/2020-10/2020 | Private Clinics | Digital monitoring of TB treatment adherence for differentiated care in private provider settings. |
| South Africa | evriMED | DS-TB | 2019-2020 | 03/2020-05/2020 | Public Clinics | Randomized control trial comparing passive evriMED box v. real time monitoring of evriMED and differentiated care based on dosing histories and level of missed doses. |
| Philippines | 99DOTS | DS-TB | 2019-2020 | 11/2019-02/2020 | Private Clinics | 99DOTS as a platform for quality TB treatment by private providers where 50% of patients in the country seek care. |
| Tanzania | 99DOTS | DS-TB | 2019-2020 | 10/2019-03/2020 | Public Clinics | Empowering TB patients in a mining population using treatment adherence technologies |
| Ukraine | evriMED | DS-TB and DR-TB | 2019-2020 | 09/2019-10/2019 | Public Clinics | Practicality, cost, and impact of combining real-time evriMED monitoring for enhanced counseling of DS-TB and DR-TB patients. |
| Uganda | 99DOTS | DS-TB | 2019-2020 | 12/2019-02/2020 | Public Clinics | From DAT to DAT: An evaluation of 99DOTS to improve TB adherence in Uganda |
