# Supplementary material for: Acceptability and feasibility of digital adherence technologies for drug-susceptible tuberculosis treatment supervision: A meta-analysis of implementation feedback
Source: PLOS Digit Health. 2023 Aug 15;2(8):e0000322. doi: 10.1371/journal.pdig.0000322 (PMC10426983; doi:10.1371/journal.pdig.0000322)
Supplement: S4 Table — (DOCX) [file pdig.0000322.s004.docx]

**S4 Table: TB REACH Wave 6 digital adherence technology projects included**

| **Project** | **DAT** | **Type of population** | **Quantitative survey results** | | **Qualitative (open-ended) survey results** | |
| --- | --- | --- | --- | --- | --- | --- |
|  |  |  | **People with TB** | **Health care workers** | **People with TB** | **Health care workers** |
| Ukraine (PATH) | evriMED | Both | X | X | X | X |
| Philippines (KNCV) | 99DOTS | Susceptible | X | X | X | X |
| Tanzania (KNCV) | 99DOTS | Susceptible | X | X | X | X |
| Uganda (UCSF) | 99DOTS | Susceptible | X | X |  |  |
| Bangladesh (icddr,b) | 99DOTS | Susceptible | X | X |  |  |
| South Africa (Aurum) | evriMED | Susceptible | X |  |  |  |
