# Supplementary material for: Acceptability and feasibility of digital adherence technologies for drug-susceptible tuberculosis treatment supervision: A meta-analysis of implementation feedback
Source: PLOS Digit Health. 2023 Aug 15;2(8):e0000322. doi: 10.1371/journal.pdig.0000322 (PMC10426983; doi:10.1371/journal.pdig.0000322)
Supplement: S5 Table — (DOCX) [file pdig.0000322.s005.docx]

**S5 Table: People with TB and health care workers included in qualitative analyses**

| **Country** | Ukraine | Tanzania | Philippines |
| --- | --- | --- | --- |
| **DAT evaluated** | evriMED | 99DOTS | 99DOTS |
| **Number of people with TB** | 19 | 200 | 106 |
| **Number of HCW** | 20 | 21 | 12 |
