# Supplementary material for: Acceptability and feasibility of digital adherence technologies for drug-susceptible tuberculosis treatment supervision: A meta-analysis of implementation feedback
Source: PLOS Digit Health. 2023 Aug 15;2(8):e0000322. doi: 10.1371/journal.pdig.0000322 (PMC10426983; doi:10.1371/journal.pdig.0000322)
Supplement: S6 Table — (DOCX) [file pdig.0000322.s006.docx]

**S6 Table. Demographic characteristics of people with TB included in qualitative analysis**

|  |  | Total  n (%)  (n=325) | 99DOTS  n (%)  (n=306) | EvriMED  n (%)  (n=19) |
| --- | --- | --- | --- | --- |
| Country |  |  |  |  |
|  | Ukraine | 19 (5·8) | n/a | 19 (100) |
|  | Tanzania | 200 (61·5) | 200 (65·4) | n/a |
|  | Philippines | 106 (32·6) | 106 (35·6) | n/a |
| Male | | 225 (69·2) | 215 (70·3) | 10 (52·6) |
| age |  |  |  |  |
|  | 15 to 44 | 199 (61·2) | 188 (61·4) | 11 (57·9) |
|  | 45 to 64 | 101 (31·1) | 94 (30·7) | 7 (36·8) |
|  | 65 or older | 25 (7·7) | 24 (7·8) | 1 (5·3) |
