# Supplementary material for: Acceptability and feasibility of digital adherence technologies for drug-susceptible tuberculosis treatment supervision: A meta-analysis of implementation feedback
Source: PLOS Digit Health. 2023 Aug 15;2(8):e0000322. doi: 10.1371/journal.pdig.0000322 (PMC10426983; doi:10.1371/journal.pdig.0000322)
Supplement: S7 Table — (DOCX) [file pdig.0000322.s007.docx]

**S7 Table. Demographic characteristics of health care workers included in qualitative analysis**

|  |  | Total  n (%)  (n=53) | 99DOTS n (%) (n=33) | EvriMED  n (%)  (n=20) |
| --- | --- | --- | --- | --- |
| Country |  |  |  |  |
|  | Ukraine | 20 (38) | n/a | 20 (100) |
|  | Tanzania | 21 (39) | 21 (64) | n/a |
|  | Philippines | 12 (22) | 12 (36) | n/a |
| Female | | 34 (63) | 17 (52) | 17 (85) |
| age |  |  |  |  |
|  | 18 to 44 | 31 (58·5) | 20 (60·6) | 11 (55·0) |
|  | 45 to 64 | 20 (37·7) | 13 (39·4) | 7 (35·0) |
|  | 65 or older | 2 (3·8) | 0 (0) | 2 (10·0) |
| Occupation |  |  |  |  |
|  | Doctor | 19 (35) | 6 (18) | 13 (62) |
|  | Nurse | 21 (39) | 16 (48) | 5 (25) |
|  | Counselor | 2 (4) | 2 (6) | 0 (0) |
|  | Pharmacist | 1 (2) | 1 (3) | 0 (0) |
|  | Other | 10 (19) | 8 (24) | 2 (10) |
