# Supplementary material for: Acceptability and feasibility of digital adherence technologies for drug-susceptible tuberculosis treatment supervision: A meta-analysis of implementation feedback
Source: PLOS Digit Health. 2023 Aug 15;2(8):e0000322. doi: 10.1371/journal.pdig.0000322 (PMC10426983; doi:10.1371/journal.pdig.0000322)
Supplement: S8 Table — (DOCX) [file pdig.0000322.s008.docx]

**S8 Table. Experience using digital adherence technologies among health care workers**

|  |  | Total  n (%)  (n=90) | 99DOTS  n (%)  (n=70) | | | EvriMED  n (%)  (n=20) | | |
| --- | --- | --- | --- | --- | --- | --- | --- | --- |
| PEOPLE WITH TB seen PER DAY | |  |  | | |  | | |
|  | 1 to 2 | 8 (8·9) | 7 (10·0) | | | 1 (5·0) | | |
|  | 3 to 5 | 34 (37·8) | 28 (40·0) | | | 6 (30·0) | | |
|  | 6 to 10 | 18 (20·0) | 16 (22·9) | | | 2 (10·0) | | |
|  | 10 or more | 30 (33·3) | 19 (27·1) | | | 11 (55·0) | | |
| Receives data from DAT | | 90 (100) | | 70 (100) | | | 20 (100) | |
| Data frequency ^a^ | |  | | |  | | |  |
|  | Daily | 73 (90·1) | 56 (91·8) | | | 17 (85·0) | | |
|  | 3 to 5 times per week | 7 (8·6) | 5 (8·2) | | | 2 (10·0) | | |
|  | Less than 3 times per week | 1 (1·2) | 0 (0·0) | | | 1 (5·0) | | |
| Data type | |  |  | | |  | | |
|  | Phone message | 50 (55·6) | 39 (55·7) | | | 11 (55·0) | | |
|  | Mobile app | 39 (43·3) | 23 (32·9) | | | 16 (80·0) | | |
|  | Computer | 12 (13·3) | 7 (10·0) | | | 5 (25·0) | | |
|  | Tablet | 18 (20·0) | 18 (25·7) | | | 4 (20·0) | | |
|  | Call from a technician | 9 (10·0) | 9 (12·9) | | | 0 (0··0) | | |
| Ways that adherence was assessed | |  | |  | | |  | |
|  | Ask when they refill meds | 51 (56·7) | 41 (58·6) | | | 10 (50·0) | | |
|  | Count pills | 21 (23·3) | 21 (30·0) | | | 0 (0·0) | | |
|  | Ask them to come to clinic | 12 (13·3) | 4 (5·7) | | | 8 (40·0) | | |
|  | Talk to family | 44 (48·9) | 32 (45·7) | | | 12 (60·0) | | |
|  | Talk on the phone | 56 (62·2) | 37 (52·9) | | | 19 (95·0) | | |
|  | Use mobile app | 77 (85·6) | 63 (90·0) | | | 14 (70·0) | | |
| Not able to access DAT to assess adherence | | 47 (52.2) | | | 41 (58·6) | | 6 (30·0) | |
| Reasons Why | |  |  | | |  | | |
|  | No electricity | 5 (10·6) | 4 (9·8) | | | 1 (16·7) | | |
|  | Poor network connection | 38 (80·9) | 34 (82·9) | | | 4 (66·7) | | |
|  | App was not working | 34 (72·3) | 33 (80·5) | | | 1 (16·7) | | |
|  | Did not want to use DAT | 0 (0·0) | 0 (0·0) | | | 0 (0·0) | | |
|  | Limited access to records | 8 (17·0) | 8 (19·5) | | | 0 (0·0) | | |

^a^ Data missing for 8 HCWs in the Philippines, 1 HCW in Tanzania
