# Supplementary material for: Acceptability and feasibility of digital adherence technologies for drug-susceptible tuberculosis treatment supervision: A meta-analysis of implementation feedback
Source: PLOS Digit Health. 2023 Aug 15;2(8):e0000322. doi: 10.1371/journal.pdig.0000322 (PMC10426983; doi:10.1371/journal.pdig.0000322)
Supplement: S9 Table — (DOCX) [file pdig.0000322.s009.docx]

**S9 Table. Internal consistency of survey responses among people with TB**

|  | Capability* | Opportunity* | Motivation* |
| --- | --- | --- | --- |
| 99DOTS | 0·45 | 0·58 | 0·54 |
| evriMED | 0·21 | 0·53 | 0·55 |
| Overall | 0·38 | 0·36 | 0·56 |

** Cronbach’s alpha is shown in the data cells*
