# Supplementary material for: Acceptability and feasibility of digital adherence technologies for drug-susceptible tuberculosis treatment supervision: A meta-analysis of implementation feedback
Source: PLOS Digit Health. 2023 Aug 15;2(8):e0000322. doi: 10.1371/journal.pdig.0000322 (PMC10426983; doi:10.1371/journal.pdig.0000322)
Supplement: S10 Table — (DOCX) [file pdig.0000322.s010.docx]

**S10 Table. Internal consistency of survey responses among health care workers**

|  | Capability* | Opportunity* | Motivation* |
| --- | --- | --- | --- |
| 99DOTS | 0·64 | 0·61 | 0·30 |
| evriMED | 0·47 | 0·74 | 0·62 |
| Overall | 0·58 | 0·70 | 0·50 |

* Cronbach’s alpha is shown in the data cells
